# Budget impact analysis of surfactant therapy for bronchiolitis in critically ill infants: the Colombian National Health System perspective

**DOI:** 10.1186/s12913-021-06347-x

**Published:** 2021-04-13

**Authors:** Jefferson Antonio Buendía, Diana Guerrero Patiño

**Affiliations:** 1 Department of Pharmacology and Toxicology, Facultad de Medicina, School of Medicine, Research Group in Pharmacology and Toxicology (INFARTO), Universidad de Antioquia, Carrera 51D #62-29, Medellín, Colombia; 2 Hospital Infantil Concejo de Medellin, Medellin, Colombia

**Keywords:** Surfactant, Colombia, Acute bronchiolitis, Budget impact analysis

## Abstract

**Background:**

Severe bronchiolitis requiring mechanical ventilation was associated with an absence of surfactant activity and phosphatidylglycerol, causing airway obstruction in acute bronchiolitis. Exogen surfactant in mechanically ventilated infants decreased duration of stay in the intensive care unit and had favorable effects on oxygenation and carbon dioxide removal. This study aimed to evaluate the budget impact of surfactant therapy for bronchiolitis in critically ill infants in Colombia.

**Methods:**

Budget impact analysis was performed to estimate the economic impact of surfactant therapy (ST) for the treatment of infants with a diagnosis of bronchiolitis, requiring mechanical ventilation. The analysis considered a 4-year time horizon and Colombian National Health System perspective. The model estimated drug costs associated with current scenario using humidified oxygen or adrenaline nebulization, and new scenario adding exogen surfactant. The size of the target population was calculated using epidemiological national data. Univariate one-way sensitivity analyses and scenario analyses were performed.

**Results:**

In the base-case analysis the 4-year costs associated to ST and no-ST were estimated to be US$ 55,188,132 and US$ 55,972,082 respectively, indicating savings for Colombian National Health equal to US$ 783,950 if ST is adopted for the routine management of patients with bronchiolitis requiring mechanical ventilation. In the one-way sensitivity analysis, only increases in the cost of the surfactant drug and cost or length of stay in the pediatric intensive unit reduce the potential savings of ST.

**Conclusion:**

ST was cost-saving in emergency settings for treating infants with severe bronchiolitis requiring mechanical ventilation. This shift in treatment approach proved to be economically favorable in the Colombian context.

## Background

Acute Respiratory Distress Syndrome represents a severe form of respiratory failure both for adults and children, with prevalence (range 2.0–12.8%) and mortality (range 18–27%) in paediatrics [1]. The case-fatality rate by severe bronchiolitis in the first two years of life is usually 0.5 to 1.5% in high-income countries and 1.74% in middle-income countries such as Latin-American [1, 2]. In Colombia, 260,873 years of life (CI 95% 208,180-347,023) were lost due to respiratory sincitial virus bronchiolitis in Colombian children under two years [3], with a high economic burden on the health system [4]. Severe bronchiolitis requiring mechanical ventilation was associated with an absence of surfactant activity and phosphatidylglycerol, causing airway obstruction in acute bronchiolitis [5]. Exogenous surfactant administration appears to change the hemodynamics of the lungs favorably and may be a promising therapy for severe bronchiolitis. A Cochrane systematic review shows that Surfactant used for mechanically ventilated infants and children with bronchiolitis decreased duration of stay in the intensive care unit and had favorable effects on oxygenation and carbon dioxide removal. The pooled mean duration of mechanical ventilation (hours) in the intervention groups was 63.04 95% confidence interval (CI) -130.43 to 4.35 h) lower in the three randomized clinical trials included in this review [6]. We hypothesize that TS can reduce the costs of care in patients with severe bronchiolitis., an aspect of vital importance in developing countries with limited health budgets. This study aimed to evaluate the budget impact of surfactant therapy (ST) for bronchiolitis in critically ill infants in Colombia.

## Methods

### Analytical framework

A budget impact analysis was performed to evaluate the potential financial impact deriving from Surfactant therapy in the market of available treatments for bronchiolitis, requiring mechanical ventilation. The analysis considered the perspective of the Colombian National Health System and was conducted over a 4-years’ time horizon. A budget impact model (BIM) was developed as a Microsoft Excel® macro-enabled workbook to evaluate the incremental budget impact of Surfactant therapy for treatment of severe bronchiolitis, requiring mechanical ventilation. The incremental budget impact was calculated by subtracting the cost of the new treatment, in which Surfactant therapy (adding to humidified oxygen or adrenaline nebulization) is reimbursed, from the cost of the conventional treatment without Surfactant therapy (only humidified oxygen or adrenaline nebulization). Full details of all assumptions used to develop the base case analysis are provided in Table 1.

**Table 1:** Assumption used to develop the base case analysis

Assumption	References
For all products compliance is considered to be 100%	Assumption based on the opinion of clinical experts
The incidence of adverse events is equal between patients treated with ST and without ST	[6]
The incidence of bronchiolitis is stable over the time horizon	Assumption based on the opinion of clinical experts
The incidence of bronchiolitis requiring mechanical ventilation is stable over the time horizon	Assumption based on the opinion of clinical experts
The market share for different types of ST are stable over the time horizon	Assumption based on the opinion of clinical experts

### Target population

The target population is represented by infants (0 to 12 months of age), term newborn without cardiac or neurological or respiratory or another chronic disease, in pediatric intensive care unit (PICU) with a diagnosis of bronchiolitis, requiring mechanical ventilation. The size of the population for the first year was calculated applying data about the total population under 2 years of age in Colombia [7], incidence of bronchiolitis in Colombia [8], frequency of infant with bronchiolitis requiring mechanical ventilation in Colombia [4, 9]. An annual population growth of 0.8% was assumed considering the average national growth rate from the period 2015–2019 [7], Table 2.

**Table 2:** Parameter used in the case base

Type of parameter	Base Case value	Range for one-way sensitivity analyses	Reference
**Demographics**			
Population	2,327,222		[7]
Annual population growth	0.8%		[7]
Pediatric weight	6.7 kg,		[10–12]
**Epidemiology**			
Bronchiolitis incidence	6.1%	4–8%	[8]
% bronchiolitis requiring mechanical ventilation	7.1%	2–10%	[4, 9]
**Cost**			
Survanta® (unit 25 mg), US$	13	10–15	[13]
Curosurf® (unit 240 mg), US$	37	30–40	[13]
Infasurf® (unit 105 mg), US$	13	10–20	[13]
**Market Share**			
Survanta® (unit 25 mg)	47.3%	40–57%	[14]
Curosurf® (unit 240 mg)	49.2%	45–55%	[14]
Infasurf® (unit 105 mg)	3.4%	2–6%	[14]

### Intervention

Exogen Surfactant (intratracheal administration of Surfactant). The information on effectiveness was extracted from the only Cochrane systematic review published which includes information from three controlled clinical trials [6]. In this review, the duration of the intensive care unit (ICU) stay was less in the ST than the control group: MD -3.31, 95% CI -6.38 -0.25 days. Serious adverse effects were not reported in these studies. Respect to the drug administration in these trials:

- The Luchetti 1998 included infants on mechanical ventilation for 24 h without significant improvement of their clinical status (uncorrected congenital heart disease and neuromuscular diseases were excluded by two studies). In this study, porcine surfactant (Curosurf) 50 mg/kg was administered in two to three doses through an endotracheal tube [10].
- Luchetti 2002 included infants who had mechanical ventilation for at least 12 h without significant improvement (uncorrected congenital heart disease and neuromuscular diseases were excluded by two studies). In this study, porcine surfactant (Curosurf) 50 mg/kg was administered in two aliquots over about five minutes through an endotracheal tube [11].
- The Tibby 2000 study enrolled infants who were ventilated for less than 24 h with an oxygenation index above five and a ventilation index above 2 (the study did not exclude children with chronic lung disease and prematurity history). In this study, bovine surfactant (Survanta) was administered in two doses (100 mg/ kg), 24 h apart, through an endotracheal tube [12].

According to the Colombian market at the time of the analysis, we have 3 types of surfactant: Survanta®, Curosurf® and Infasurf® have a market shares of 47.35%, 49.20 and 3.4% respectively [14]. The cost of treatment per patient was calculated:

- the total amount of milligrams per patient/treatment was estimated for each types of surfactant according to the dosing schedules mentioned above, and assuming a patient with average weight of 6.7 kg,
- The cost of each types of surfactant per patient was estimated by multiplying the cost per milligram of each types of surfactant, obtained from the national list of drug costs [13], by the total amount of milligrams estimated per patient/treatment calculated in the previous step
- The final cost of treatment per patient was obtained from the sum of the results of the cost of each type of surfactant per patient multiplied by their market share.

We assumed to progressively gain market sales from surfactant therapy. In the base-case scenario the uptake rate of surfactant therapy was assumed to be 25%, increasing to 25% each year respectively, according to he estimates of the marketing authorization holder and recommendation of national guidelines for economic evaluations in Colombia [15].

### Time horizon

The time horizon defined was four years. The maximum follow-up time was set to be four years. A longer perspective was not considered relevant for the budget holder following recommendation of national guidelines for economic evaluations in Colombia [15]. All results are depicted cumulatively from 1 to 4 years.

### Resource use and cost

The costs of each outcome defined previously were estimated directly from medical invoices and electronic medical records of 193 infants admitted in tertiary centers in Rionegro, Colombia, with a diagnosis of bronchiolitis, according to the national clinical guidelines of bronchiolitis. This cost and clinical characteristics of these patients were published previously [4]. Brief, the direct costs considered in the analysis include medical consultation at the emergency room, specialist referrals, chest physiotherapy, diagnosis support (laboratory, electrocardiogram, x-ray, etc.), medication (oxygen, nebulization, antibiotics, corticosteroids, bronchodilators, etc.), medical devices, hotel services in the intensive care unit, and hotel services in the general medical ward. All treatment costs include the administration and preparation costs covered by the treating organization. All adverse events were assumed to be fully reversible and thus not to cause any additional costs to the hospital district. To avoid data errors during medical record abstraction, we used software (Excel MS®) with automatic calculation functions and error alerts and a review of outliers by the research team. We used US dollars (currency rate in 2019: US$ 1.00 = COP$ 3000) [16] to express all costs in the study. For the valuation of the indirect costs associated with the loss of parents’ productivity, the human capital method was used, assuming everyone receives an income of at least a legal minimum wage for formal or informal work. The cost-opportunity of the productivity loss at the workplace and the caregiver was assessed based on the minimum wage without including transportation assistance (US$ 229.81 per month). The legal minimum wage approved by the government was taken as a reference and not an average or median wage thereof, given that in Colombia, over 75% of the population has this value as their income [7]. Because all patients with acute asthma episodes included in this study were children, we assumed that at least one family member accompanied the patient permanently during hospitalization, as pediatric hospitals in the country usually allow only one companion per patient in the hospital. The cost associated with transportation and food (does not include a stay), was assumed to correspond to 50% of minimum wage per day.

### Sensitivity analyses

The robustness of the base-case was evaluated with one-way sensitivity analyses. The parameters used in the analysis were varied once as detailed in Table 3. Expert opinion and literature data were used to determine ranges of parameters to be tested in the sensitivity analysis. Results of the sensitivity analysis are presented in a tornado diagram showing the impact on base-case of uncertainty in the parameters used in the model. Microsoft Excel® was used in all analyses.

**Table 3:** Cost (US$) used in base case and sensitivity analyses

Model input	Base case value	SA range for one-way sensitivity analyses	Distribution
**Intervention cost**			
ST per patient day	203	43–352	γ (SD:197)
**Hospitalization cost**			
Daily cost in pediatric ward	48.82	47,64 50.00	γ (SD:3,20)
Hospital length of stay (days)	5,8	4,00-6,01	γ (SD:2,03)
**PICU related cost**			
Daily cost in PICU	327,35	326,26–328-43	γ (SD:5,49)
Reduction in PICU lenght of stay (days) by ST	3.31	0.25–6.38	γ (SD:1.8)
**Emergency visit prior hospitalization cost**			
Daily cost of emergency ward	12,83	12,19-13,46	γ (SD:3,20)
**Direct medical cost per patient-day**			
Specialist referrals	10,67	10,31-11,01	γ (SD:1,72)
Chest physiotherapy	5,15	4,90-5,39	γ (SD:1,23)
Chest radiography	2,84	2,70-2,98	γ (SD:0,73)
Others diagnostic imaging	0,01	0,0-0,022	γ (SD:0,08)
Complete blood cell counts	1,12	1,05-1,17	γ (SD:0,28)
RSV test	2,71	2,83-3,03	γ (SD:2,72)
Other laboratory tests	4,40	4,23-4,47	γ (SD:0,37)
Oxygen	1,37	1,28-1,45	γ (SD:0,41)
Nebulization	16,23	1,28-1,45	γ (SD:4,52)
LEV	1,10	1,07-1,13	γ (SD:0,16)
Antibiotics systemics	1,21	1,11-1,30	γ (SD:0,49)
Systemic o Inhaled Corticosteroids	0,08	0,0-0,90	γ (SD:4,18)
Bronchodilators	0,04	0,03-0,04	γ (SD:0,02)
Other drugs	0,65	0,60–0,68	γ (SD:0,04)
Medical devices	10,24	9,71-10,76	γ (SD:2,66)
**Indirect cost patient-day**	17,24	16.38–18,07	γ (SD:4,30)

## Results

### Base-case results

**Fig. 1:**
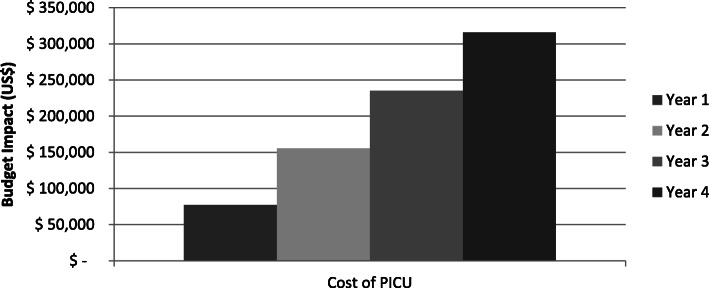
Savings in pediatric intensive critical unit (PICU) cost

In the base-case analysis the 4-year costs associated to ST and no-ST were estimated to be US$ 55.188.132 and US$ 55.972.082 respectively, indicating savings for Colombian National Health equal to US% 783.950 if ST is adopted for the routine management of patients with bronchiolitis requiring mechanical ventilation (Table 4). The savings by ST increased over the years due to the greater number of patients progressively receiving ST and savings of cost of PICU, Fig. 1.

**Table 4:** Budget impact

	Overall num patients	Num patients treated with ST	Cost ST (US$)	Cost No ST (US$)	Savings (US$)	% Savings
**Year 1**	3851	963	13.747.011	13.824.148	77.138	0,56%
**year 2**	3882	1941	13.780.599	13.936.124	155.525	1,12%
**Year 3**	3914	2935	13.813.829	14.049.006	235.177	1,67%
**Year 4**	3946	3946	13.846.694	14.162.803	316.110	2,23%
**Total**	15,593	9785	55.188.132	55.972.082	783.950	1,40%

**Fig. 2:**
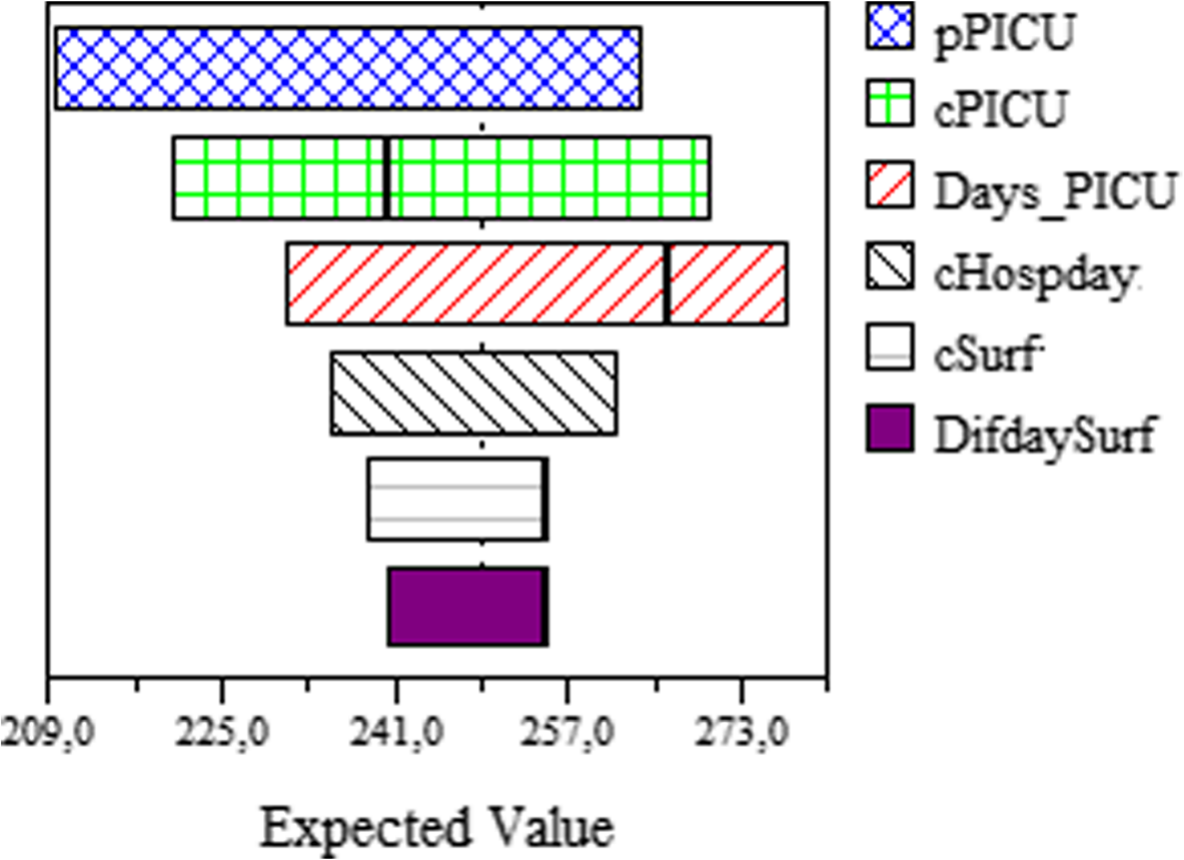
Tornado diagram

**Fig. 3:**
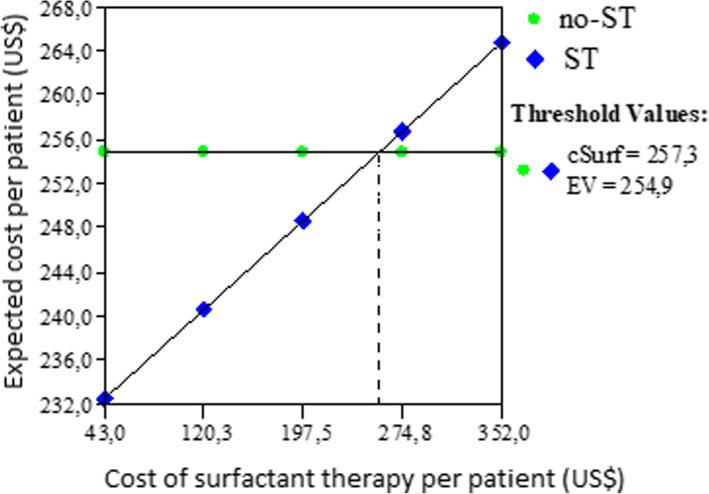
Threshold analysis of cost of surfactant therapy

The Tornado diagram shows the results of one-way sensitivity analysis, Fig. 2.Expected cost of ST was always lower than no ST except in three variables: drug cost of surfactant and daily cost in PICU and PICU length of stay, Fig. 2. A increase in the total cost of treatment with ST higher than U$257 per patient, resulted in expected cost per patient in ST scenario more elevated than no ST scenario (US$ 259 vs US$ 254), see Fig. 3. When the PICU length of stay was higher than 9.4 days, also the expected cost per patient in ST scenario was higher than no ST and if the daily cost in PICU was higher than US$ 266 the expected cost per patient in ST scenario was higher than no ST, losing also in both cases the savings generated by the population by the use of ST. In scenarios where the expected cost per patient or the PICU length of stay are lower, ST will be cost-saving.

## Discussion

Our study suggests that ST is a cost-saving for the treatment of infant with severe bronchiolitis requiring mechanical ventilation. The therapy only with humidified oxygen or adrenaline nebulization without ST proved to be the most expensive therapy, being associated with the highest total cost. Treatment with ST over four years was cost-saving thanks to the fewer cost of mechanical ventilation being observed with this regimen. Compared with the current therapy without ST, the alternative treatment with MS provided total cost saving US $ 771.377..

Previous publications have shown that bronchiolitis has a relevant impact on healthcare resources consumption and related expenditures, especially in severe cases, and any intervention that reduces the burden of disease and costs is highly appropriate to any health system [3, 4, 9, 17]. Surfactant is the therapy with the highest level of expectations due to a positive effect in PICU length of stay, but still a few evidence in endpoints such as mortality. A recent study shows that the use of porcine Surfactant improves oxygenation, P/F ratio, and pH in a population of children with moderate or severe pediatric acute respiratory distress syndrome caused by multiple diseases [18]. In an infant with severe RSV infection, the administration in mechanically ventilated infants with acute respiratory failure due to RSV bronchiolitis improves gas exchange and respiratory and shortens the duration of mechanical ventilation and PICU stay compared with control [6]. Despite that, the duration of mechanical ventilation was no different in the surfactant group compared to the control group (mean difference (MD) -63.04 h, 95% confidence interval (CI) -130.43 to 4.35), there was a trend towards beneficial effects of Surfactant [6]. It is possible that with more randomized controlled trials, this effect could be reached. Also, this systematic review reported that no adverse hemodynamic effects (no significant change in heart rate and blood pressure) and no complications were noted in the clinical trials included in this publication. We can conclude, the ST would be a therapeutic option safe, with positive clinical effects in the PICU length of stay and in health cost [19].

The total health spending for communicable diseases in Colombia ranges from $ 70 to $ 80 million annually [20]. If this drug is adopted within the clinical practice guidelines, of the total mentioned above, almost 1 million dollars could be saved annually;. Otherwise, the ST was always the cost-saving strategy in all ranges of probabilities of requiring mechanical ventilation evaluated. A relevant result was to find a drug cost of the ST per patient at which this therapy does not cost-saving. This threshold (US$ 257) can be used as a reference for the control and regulation of prices in the country. Show evidence related to the economic impact this drug is essential to policy makers and physician [21] especially in developing countries where economic evaluation of drugs and medical devices in pediatric patients are increasing [17, 22–25].

Our study has some limitations. We used retrospective data reported in a previously cost-effectiveness study, and this information does not exclude the possibility that medical invoices were incomplete or missing data. This study reported that several measures were employed to ensure data accuracy, including software with automatic calculation functions and error alerts and a review of outliers by the research team. Another limitation in the design was to the assumption of complete adherence of this therapy, which can reduce the budget impact of ST. A clinically significant reduction can be seen with outpatient medications, but with medications in critically ill patients, high adherence can be expected.

In conclusion, ST was cost-saving in emergency settings for treating infants with severe bronchiolitis requiring mechanical ventilation. This shift in treatment approach proved to be economically favorable, reducing the cost of hospitalization and mechanical ventilation. This evidence can be used by decision-makers in our country to improve clinical practice guidelines and should be replicated to validate their results in other middle-income countries.

## Data Availability

The raw data supporting your findings can be request to CIEMTO (http://ciemto.medicinaudea.co/).

## Abbreviations

ST: Surfactant therapy
no-ST: Control or non-surfactant therapy
BIM: Budget impact model
PICU: Pediatric intensive care unit

## References

[1] Khemani RG, Smith LS, Zimmerman JJ, Erickson S, Pediatric acute lung injury consensus conference G (2015). Pediatric acute respiratory distress syndrome: definition, incidence, and epidemiology: proceedings from the pediatric acute lung injury consensus conference. Pediatr Crit Care Med.

[2] Word Bank The Word Bank In Middle Income Countries. Available from: https://www.worldbank.org/en/country/mic. Accessed 07 Apr 2021.

[3] Villamil JPS, Polack FP, Buendía JA (2020). Disability-adjusted life years for respiratory syncytial virus in children under 2 years. BMC Public Health.

[4] Buendia JA, Patino DG. Costs of Respiratory Syncytial Virus Hospitalizations in Colombia. Pharmacoecon Open. 2020. 10.1007/s41669-020-00218-7 PMC7895874 32418086

[5] Skelton R, Holland P, Darowski M, Chetcuti PA, Morgan LW, Harwood JL (1999). Abnormal surfactant composition and activity in severe bronchiolitis. Acta Paediatr.

[6] Jat KR, Chawla D (2015). Surfactant therapy for bronchiolitis in critically ill infants. Cochrane Database Syst Rev.

[7] Departamento, Nacional, (DANE) DE. Archivo Nacional de Datos 2019 [Available from: https://sitios.dane.gov.co/anda-index/. Accessed 07 Apr 2021.

[8] Instituto, Nacional, Salud d. Infeccion respiratoria aguda en Colombia 2017 [05/07/2019]. Available from: https://www.ins.gov.co/buscador-eventos/Informesdeevento/Informe%20IRA%20Final%202017.pdf. Accessed 07 Apr 2021.

[9] Rodriguez-Martinez CE, Sossa-Briceno MP, Castro-Rodriguez JA (2019). Direct medical costs of RSV-related bronchiolitis hospitalizations in a middle-income tropical country. Allergol Immunopathol (Madr)..

[10] Luchetti M, Casiraghi G, Valsecchi R, Galassini E, Marraro G (1998). Porcine-derived surfactant treatment of severe bronchiolitis. Acta Anaesthesiol Scand.

[11] Luchetti M, Ferrero F, Gallini C, Natale A, Pigna A, Tortorolo L, Marraro G (2002). Multicenter, randomized, controlled study of porcine surfactant in severe respiratory syncytial virus-induced respiratory failure. Pediatr Crit Care Med.

[12] Tibby SM, Hatherill M, Wright SM, Wilson P, Postle AD, Murdoch IA (2000). Exogenous surfactant supplementation in infants with respiratory syncytial virus bronchiolitis. Am J Respir Crit Care Med.

[13] MinSalud. Termometro de precios de medicamentos 2014 [Available from: https://www.minsalud.gov.co/salud/MT/Paginas/termometro-de-precios.aspx. Accessed 07 Apr 2021.

[14] MinSalud. Sistema Integral de informacion de la proteccion social 2019 [Available from: https://web.sispro.gov.co/WebPublico/Consultas/ConsultarCNPMCadenaComercializacionCircu2yPA_028_2_2.aspx. Accessed 07 Apr 2021.

[15] Instituto de Evaluación Tecnológica en Salud (2014). Manual para la elaboración de análisis de impacto presupuestal.

[16] la Bd, Republica. Tasa Representativa del Mercado (TRM - Peso por dólar) 2019 [cited 2020. Available from: https://www.banrep.gov.co/es/estadisticas/trm. Accessed 07 Apr 2021.

[17] Buendia JA, Acuna-Cordero R (2020). The cost-effectiveness of hypertonic saline inhalations for infant bronchiolitis. BMC Health Serv Res.

[18] Wolfler A, Piastra M, Amigoni A, Santuz P, Gitto E, Rossetti E, Tinelli C, Montani C, Savron F, Pizzi S, D’amato L, Mondardini MC, Conti G, de Silvestri A (2019). A shared protocol for porcine surfactant use in pediatric acute respiratory distress syndrome: a feasibility study. BMC Pediatr.

[19] Sullivan SD, Mauskopf JA, Augustovski F, Jaime Caro J, Lee KM, Minchin M, Orlewska E, Penna P, Rodriguez Barrios JM, Shau WY (2014). Budget impact analysis-principles of good practice: report of the ISPOR 2012 budget impact analysis good practice II task force. Value Health.

[20] MinSalud. Estructura del gasto en Salud Pública en Colombia 2018 [Available from: https://www.minsalud.gov.co/sites/rid/Lists/BibliotecaDigital/RIDE/DE/PES/estructura-gasto-salud-publica-colombia.pdf. Accessed 07 Apr 2021.

[21] Buendia JA, Zuluaga AF (2014). Physicians insight about adverse drug reaction to frequently used medication groups in Bogota (Colombia). Biomedica..

[22] Buendia JA, Sanchez-Villamil JP, Urman G (2016). Cost-effectiveness of diagnostic strategies of severe bacterial infection in infants with fever without a source. Biomedica..

[23] Antonio Buendia J, Colantonio L (2013). Costo-Efectividad de la Proteina C Reactiva, Procalcitonina y Escala de Rochester: Tres Estrategias Diagnosticas para la Identificacion de Infeccion Bacteriana Severa en Lactantes Febriles sin Foco. Value Health Reg Issues.

[24] Buendia JA, Acuna-Cordero R, Rodriguez-Martinez CE (2020). The cost-utility of intravenous magnesium sulfate for treating asthma exacerbations in children. Pediatr Pulmonol.

[25] Buendia JA, Talamoni HL. Cost-utility of use of sputum eosinophil counts to guide management in children with asthma. J Asthma. 2020:1–7. 10.1080/02770903.2020.1830412. 10.1080/02770903.2020.1830412 33026885

